# The effects of super spreading events and movement control measures on the COVID-19 pandemic in Malaysia

**DOI:** 10.1038/s41598-022-06341-1

**Published:** 2022-02-09

**Authors:** Lai Chee Herng, Sarbhan Singh, Bala Murali Sundram, Ahmed Syahmi Syafiq Md Zamri, Tan Cia Vei, Tahir Aris, Hishamshah Ibrahim, Noor Hisham Abdullah, Sarat Chandra Dass, Balvinder Singh Gill

**Affiliations:** 1 Institute for Medical Research (IMR), National Institutes of Health (NIH), Ministry of Health Malaysia, 40170 Setia Alam, Malaysia; 2 Ministry of Health Malaysia, 62590 Putrajaya, Malaysia; 3 Heriot-Watt University Malaysia, 62200 Putrajaya, Malaysia

**Keywords:** Diseases, Health care

## Abstract

This paper aims to develop an automated web application to generate validated daily effective reproduction numbers (Rt) which can be used to examine the effects of super-spreading events due to mass gatherings and the effectiveness of the various Movement Control Order (MCO) stringency levels on the outbreak progression of COVID-19 in Malaysia. The effective reproduction number, Rt, was estimated by adopting and modifying an Rt estimation algorithm using a validated distribution mean of 3.96 and standard deviation of 4.75 with a seven-day sliding window. The Rt values generated were validated using thea moving window SEIR model with a negative binomial likelihood fitted using methods from the Bayesian inferential framework. A Pearson’s correlation between the Rt values estimated by the algorithm and the SEIR model was r = 0.70, p < 0.001 and r = 0.81, p < 0.001 during the validation period The Rt increased to reach the highest values at 3.40 (95% CI 1.47, 6.14) and 1.72 (95% CI 1.54, 1.90) due to the Sri Petaling and Sabah electoral process during the second and third waves of COVID-19 respectively. The MCOs was able to reduce the Rt values by 63.2 to 77.1% and 37.0 to 47.0% during the second and third waves of COVID-19, respectively. Mass gathering events were one of the important drivers of the COVID-19 outbreak in Malaysia. However, COVID-19 transmission can be fuelled by noncompliance to Standard Operating Procedure, population mobility, ventilation and environmental factors.

## Introduction

The novel Severe Acute Respiratory Syndrome Coronavirus 2 (SARS-CoV-2) which was first discovered in Wuhan, China, late in December 2019 was declared a pandemic by the World Health Organization (WHO) in 2020^1^. As of 8 October 2021, more than 237 million cases have been reported with over 4.8 million deaths worldwide^2^. As the COVID-19 pandemic progresses, several countries globally have reported super spreading events that have attributed to the exponential rise in COVID-19 cases^3,4^. These events are likely to cause the observed sudden surge of cases due to increase disease transmission at mass gatherings and overcrowding events at particular localities^3–5^. In addition, infected individuals with higher disease transmissibility would further increase the risk of larger outbreaks^6^. Mass gatherings including political events, conferences, and sporting events are known to be super spreading events that have resulted in large COVID-19 outbreaks in several countries^6–8^.

In Malaysia, the first wave of COVID-19 that lasted for a month started as a result of imported cases on 25 January 2020 and reported a total of 22 cases^9^. A much larger second wave started on 27 February 2020, following a 4-day mass gathering at Sri Petaling, Kuala Lumpur^10^. This mass gathering resulted in a massive transmission of COVID-19 infection in Malaysia which subsequently caused the largest COVID-19 cluster during the second wave with 3375 confirmed cases with 34 deaths reported^11,12^. Currently, Malaysia is facing the third COVID-19 wave which was triggered by the Sabah state electoral process on 26 September 2020 resulting in several mass gatherings and population movements^13^.

In response to the COVID-19 pandemic, the Malaysian government instituted various Non-Pharmaceutical Interventions (NPIs), one of them being the Movement Control Order (MCO) which was first implemented during the second wave on 18 March 2020^14^. The MCO aimed to flatten the epidemic curve and reduce disease transmissibility by enforcing movement restrictions and the prohibition of gatherings^15^. The strict MCO that was initially implemented was gradually relaxed accordingly with the reduction of reported cases. To strike a balance between lives and livelihood, adjustments to the stringency of the MCO are carried out through continuous assessments of the available disease transmission indicators such as incidence rate, growth rate, doubling time, and reproduction number^15^.

While indicators such as incidence rate, growth rate and doubling time may be useful for outbreak monitoring, they are unable to estimate disease transmissibility and cannot be used in forecast models. Studies have shown that estimating disease transmissibility during an epidemic, which is denoted by the reproduction number (R0), is important as it provides an aggregated measure of the transmissibility of an emerging infection with the ability to describe the progression of an epidemic, and provides a threshold of which an outbreak will either halt or continue to grow^16,17^. Therefore, reproduction numbers are effective and useful indicators for disease surveillance and control purposes^15^.

The basic reproduction number (R0) is the average number of secondary cases of infections caused by a single infected individual over an infectious period which is static over time,, On the other hand, the time-varying reproduction number (Rt) is defined as the average number of secondary cases per primary case at a particular time point^16,17^. Values of the Rt identify changes in disease transmission over different points in time as a result of disease control interventions and population immunity^16,17^. Therefore, Rt values are key to characterizing the evolution of an outbreak, assessing the effectiveness of disease control measures, be used as an early warning outbreak indicator as it can provide a threshold for which an outbreak may increase or decrease exponentially, and be used as a parameter in forecast models^18^.

COVID-19 is a novel disease that is characterized by sudden increases in transmissibility as a result of super-spreading events. Therefore, it is important to monitor the outbreak continuously (e.g., daily) to provide sensitive information on the progression of the outbreak. Similarly, the MCO measures implemented to control this outbreak require constant adjustments to strike a balance between life and livelihoods. Rt estimates can provide a sensitive indicator on the current disease transmissibility and progression of the pandemic which is important for adjusting the stringency levels of the MCO^19^. As cases of COVID-19 in Malaysia are increasing, there is a need to develop a process to estimate accurate and validated daily Rt values for Malaysia at the national and state levels daily. Hence, this paper aims to develop an automated web application to generate validated daily Rt values which can be used to examine the effects of super-spreading events due to mass gatherings and the effectiveness of the various MCO stringency levels on the outbreak progression of COVID-19 in Malaysia.

## Methodology

We measured the effects of different events on COVID-19 disease transmissibility by measuring the change and progression of the daily estimated Rt values for Malaysia and the states of Selangor and Sabah. These events include disease spreading events or disease propagation events such as large gatherings and movements of populations, and disease control events which are the MCO stringency levels during the first, second and third wave of COVID-19 outbreak in Malaysia. These MCO levels were categorized into enhanced Movement Control Order (MCO) phases 1 to 3, conditional Movement Control Order (CMCO), and recovery Movement Control Order (RMCO) which was enforced from 18 March 2020 to 29 March 2021^14^. As for disease spreading events, the duration of the event was measured as the period of the event and 14 days after to take into account the maximum incubation period of the disease for its effects to be observed, and also to factor in the effects of delay in case reporting. A 14-day incubation was selected as previous works in the literature have estimated the incubation period of COVID-19 to be anywhere between 2 and 14 days^20^.

The study period from 25 January 2020 to 29 March 2021 was selected as this study essentially focusses on the effects of super-spreading events and epidemic control measures (that is, the movement control order) during the first, second and third waves of the COVID-19 outbreak in Malaysia. However, the period after 29 March 2021 was not included in this study as it was categorized as the fourth wave where there were several changes to the dynamics of the COVID-19 outbreak in Malaysia which would not allow for accurate measurement of effects of movement control measures and mass gathering events. During the fourth wave the categorization of the movement restrictions was changed into National Recovery Plan category 1 to 4 which is not comparable to the movement control order levels during the first three waves^21^. In addition, the introduction of vaccination during the fourth wave would further have an effect on the disease dynamics and confound the effect of the super spreading events and movement control orders^21^.

Data analysis was performed using the *EpiEstim* EXCEL spreadsheet with macro function for the estimation of the Rt values^18^. R statistical software version 4.0.4 with packages such as *incidence*, *xts*, *shiny* and *jsonlite* were used for the development of the automated system for the generation of real-time daily Rt values^22^. The validation phase involved analyzing the data in R statistical software version 4.0.4^22^ which involved performing Pearson’s correlation and Bayesian regression analysis for estimating the model fit.

### Estimating Rt values

Daily Rt values were estimated from 1 February 2020 to 8 November 2020 to quantify the temporal changes in the transmission intensity of the COVID-19 outbreak in Malaysia^18^. The Rt was calculated by adopting and modifying the Rt algorithm developed by Anne Cori et al.^18^. This method produces statistically robust R values which incorporates uncertainty in the distribution of the serial interval using the earlier-mentioned *EpiEstim* EXCEL spreadsheet and calculates the Rt values using locally validated parameters^18^. The Rt values were estimated using daily case numbers sourced from the Crisis Preparedness Response Centre (CPRC), Ministry of Health Malaysia (MOH)^13^.

The parameters and input data used in this algorithm to estimate the daily Rt values are (i) prior distribution mean, (ii) standard deviation, (iii) serial intervals, and (iv) daily COVID-19 case numbers. These parameter values were obtained from literature and validated for use in the Malaysian context^23^. The generated Rt values for the first, second and third COVID-19 outbreak waves, and mass gathering events in Sri Petaling and Sabah, and the MCO phases were described in terms of the median, highest and lowest Rt values, and median Rt trends. Changes in the Rt point estimates and its 95% confidence intervals for each wave and corresponding events throughout its duration were quantified in terms of percentage increase or decrease by fitting a linear equation to the Rt trends of each wave using the EXCEL trend line function^24^. The daily percentage of increase or decrease is calculated based on the gradient of the linear equation whereby a negative gradient indicates a reducing trend and a positive gradient indicates an increasing trend^24,25^. The Rt values estimated during the first and second COVID-19 waves in Malaysia were generated using all reported cases. However, for the third wave, we estimated the Rt values using only community cases and excluded detention case clusters to specifically measure only the effects of mass gathering events and not detention clusters in the community^13^.

### Validation of parameters and Rt values

Parameter validation was carried out based on identifying the most appropriate parameter values by matching the generated Rt values using this algorithm to the R0 values obtained using the Susceptible-Exposed-Infected-Recovered (SEIR) models at specific time intervals, and subsequently, fitting the generated Rt trends to the COVID-19 cases in Malaysia. The final Rt algorithm was parametrized using a prior distribution mean of 3.96 and a standard deviation of 4.75^23^. In addition, the estimation of Rt by this algorithm requires a selection of a range of days (for the sliding window) where we attempted to use a 7- and 14-day width for the sliding window. The length of the window would affect the sensitivity of the estimates (i.e., short window would increase the sensitivity, however, would result in increased variability (noise) in the estimates). Subsequently, a seven-day sliding window was selected as it provided the best balance between sensitivity and noise^18^.

Similarly, the Rt values generated by the algorithm in this study were validated based on corresponding R0 values found using a moving window SEIR model^15^. Parameter estimates and uncertainty quantification were obtained using Bayesian methods of inference. R programming software was used to develop the codes together with observed data comprising of 55 data points of COVID-19 cases from 1 September 2020 to 25 October 2020. During the validation, a 14-day period was selected for the moving window so that the date in focus would be the central date of that window. Next, 95% and 99% equal tail credible intervals were obtained based on Bayesian posterior distributions and associated Monte Carlo computations^26^. The estimated Rt values were checked to see if they lied within each of their credible intervals, and a Pearson’s correlation analysis was performed^27^. A high percentage of coverage by the credible intervals and a significant correlation indicate that the Rt values obtained by the Rt algorithm were adequate measures for capturing the underlying disease dynamics. The output of Rt values estimated using the algorithm and outputs from the the SEIR model were also correlated using Pearson’s correlation during the second (Epidemiological week from 14 to 17) and third (Epidemiological week 40 to 43) COVID-19 waves in Malaysia. Computing Pearson’s correlation required us to check various assumptions of linear regression under which interpretation of the correlation remains valid. These are explained in detail in the next paragraph.

Prior to conducting the computations related to the Pearson’s correlation, the assumptions of linear regression which include normality, homoskedasticity and linearity were tested. Normality of data was tested using the Kolmogorov–Smirnov (K–S) and Shapiro–Wilk tests where in p-values have shown evidence in favor of distributive normality^28^. Outliers were tested using box-plots where scores either less than or greater the lower and upper fences, respectively, were identified as outliers. The lower fence was calculated as the first quartile minus 1.5 times the interquartile range whereas the upper fence was calculated as the third quartile plus 1.5 times the interquartile range^28^. All scores were within the ranges of the fences, indicating no presents of outliers.

Homoscedasticity was tested using the Spearman’s rank correlation coefficient from a linear regression analysis^29^. The Rt values from the proposed algorithm and the R0 values from the moving window SEIR model were taken to be the response and explanatory variables, respectively. Spearman’s rank correlation coefficient was calculated for the resulting residuals and the corresponding predicted values of Rt. The value of the Spearman’s correlation had a corresponding p-value which was non-significant indicating that the residuals formed a uniform band around zero in the residual scatter plot, which also indicates the adequacy of the linearity and homoscedasticity assumptions^29^. Linearity was additionally corroborated using scatter plots between the Rt estimates generated from the algorithm and SEIR model where we found no evidence of ce of coning in the scatter plot, also indicating linearity^29^. In summary, all assumptions needed for obtaining the Pearson’s correlation coefficient were satisfied. Appendix 1 shows the results of normality, identification of outliers, homoscedasticity and linearity for the data during the validation period in the second and third waves.

### Development of an automated web application system for the generation of real-time daily Rt values

To provide real-time daily Rt updates, the algorithm was automated by developing a web-based application using the R programming software with *incidence*, *xts*, *shiny* and *jsonlite* packages. This application extracts the parameters and case data from relevant sources such as the National Security Council Malaysia (MKN) and MOH COVID-19 portals^13^. Automated parsing and structuring of extracted data were done in *shiny* server-side backend processing. Sourced data from MKN/MOH portals were transformed into a time series format using the *incidence* and *xts* packages of R, and the *reactive* function provided by R’s *shiny* application allows for reactive computation of outcomes based on user input or default parameters. Data syncing and back-end computations were done to ensure the stability and ease of use of this application.

This automated process updates the latest Rt values and minimizes inconsistencies due to human error. The *reactive* function from *shiny* enables cycle updates every second through the enveloped function which refreshes the required data and computes the Rt values resulting in real-time Rt updates. This process begins by using the *jsonlite* package to reformat the data sourced from MKN/MOH portal into an R readable format that is compatible with the algorithm. Preprocessed data were then fitted into the *EpiEstim* package for calculation of the real-time Rt values.

In addition, the application allows for real-time changes in user input parameters by the continuous updating of backend data computations and reactive functions. Features developed for this application include the automated rendering of outputs, optional sliding windows size input and changes in parameter values which are used for the testing and optimization processes. An interactive interface with visual elements was also incorporated into the application to allow for smooth page rendering. These automated and reactivity functions were developed in this application to provide the most optimal user experience.

To ensure the application produces continuous daily Rt outputs and to prevent downtime, several measures were taken. Firstly, downtime was prevented by introducing redundancies through hosting the application on a cloud-based server and a backup local server. Secondly, access to COVID-19 case data and parameter values were sourced from multiple open access data sources in the event of data inaccessibility at the source portals. Thirdly, the computational processes of the application can be overridden manually as a final failsafe mechanism in the event of automation failure.

**Figure 1:**
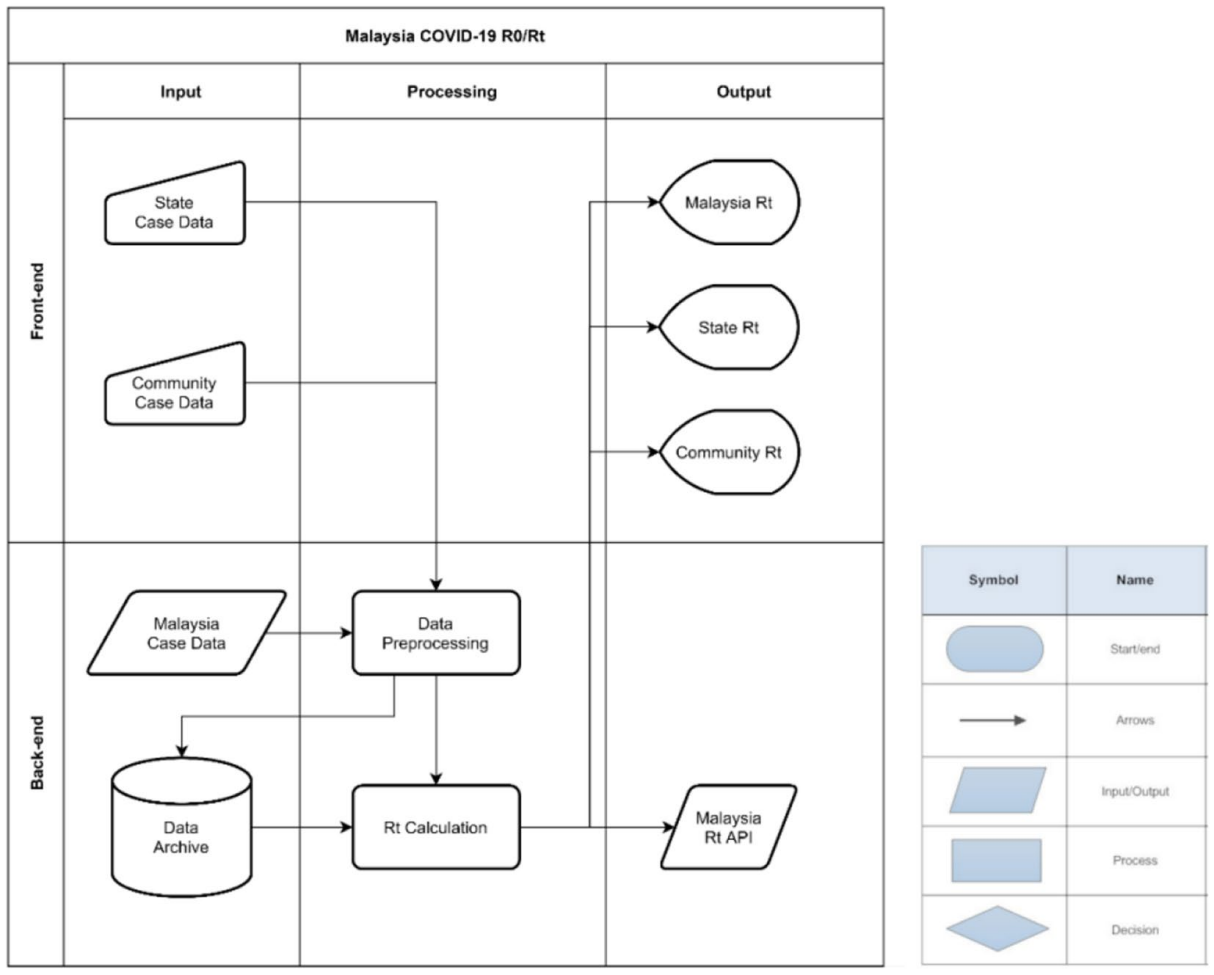
HYPERLINK "sps:id::fig1||locator::gr1||MediaObject::0" Automated web application system flow chart.

**Figure 2:**
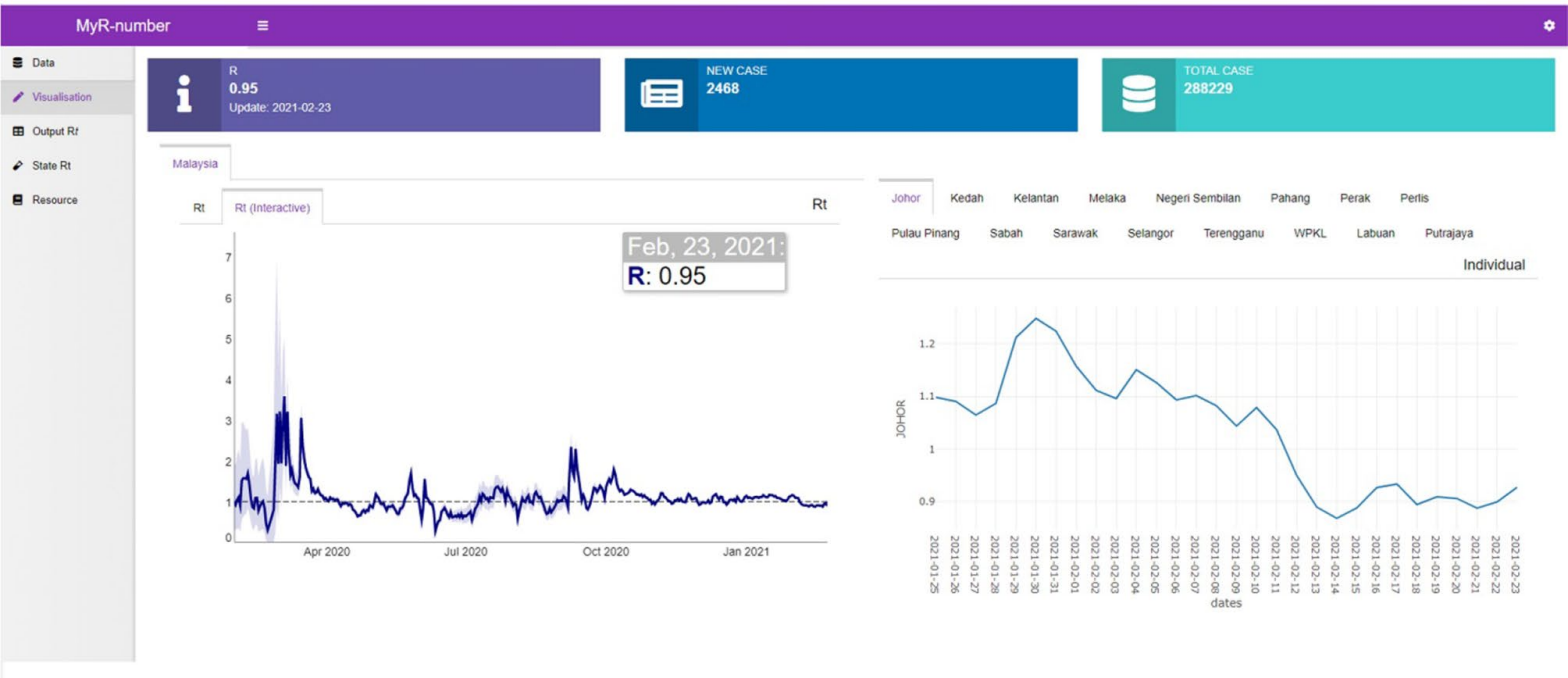
Automated web application system (auto-generated daily Rt plots at National and State levels).

The automated web application system generates the daily Rt flow using a back-end and a front-end process as shown in Fig. 1. This system enables users to monitor the daily Rt value along with their 95% confidence intervals in interactive plots (Fig. 2). The interactive real-time continuous daily Rt values are generated on a real-time basis from the availability of daily cases reported on the MOH web portal. Additionally, this system allows for interactive interphase for manual Rt calculation and adjustment to input parameters.

### Ethical declarations

The study was registered with National Medical Research Register (NMRR-21-300-58289). No ethics approval was required.

## Results

### Validation of Rt values

**Figure 3:**
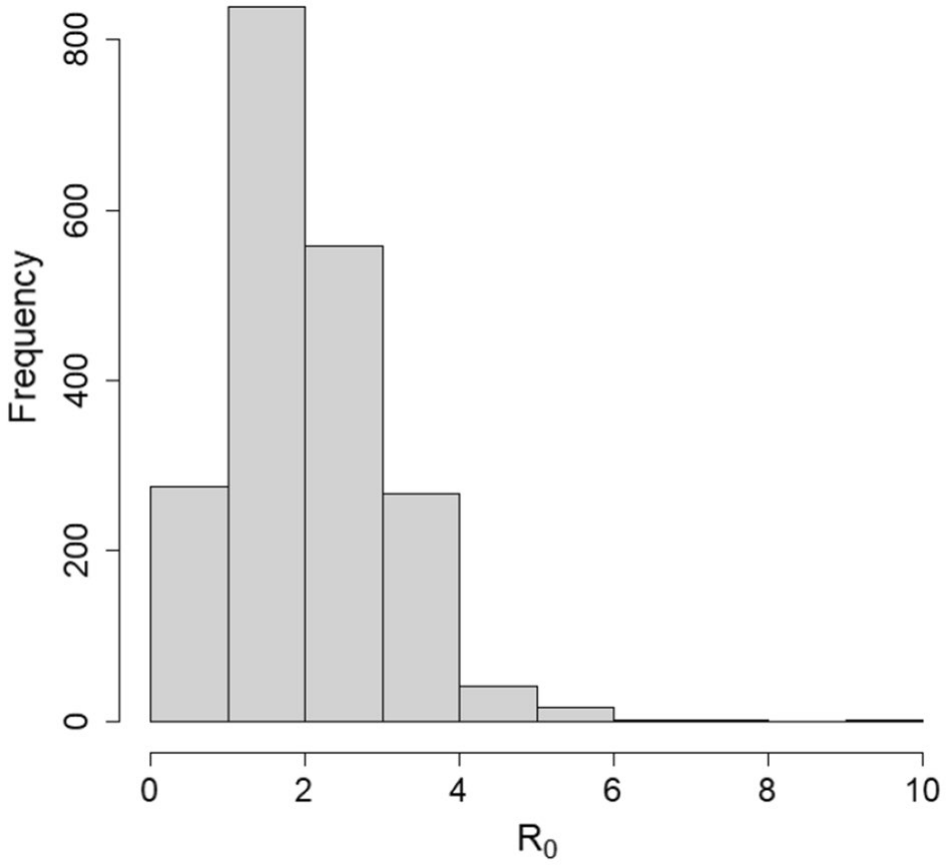
Posterior distribution of Rt obtained using the Bayesian methodology.

The validation of the Rt values generated by the algorithm was performed by comparing them with the corresponding R0 values estimated by the moving window SEIR model fitted to observed COVID-19 cases. To account for uncertainty, the validation—was performed using the posterior distribution of Rt obtained from the Bayesian inference methodology. An illustration of this methodology is provided for the date of 6 October 2020 where the Rt value of 1.78 was found by the algorithm. The resulting posterior distribution of Rt had the highest posterior frequency around the same value of 1.78 (see Fig. 3). For this date, the 95% and 99% posterior credible intervals were obtained as (0.52, 4.30) and (0.11, 6.82), respectively. The Rt value 1.78 was covered by both credible intervals. To validate all Rt values in a similar way, we found that Out of the 55 validation tests conducted, a total of 54 (98%) and 48 (87%) of them had their Rt values inside the 95% and 99% credible intervals, respectively.

**Figure 4:**
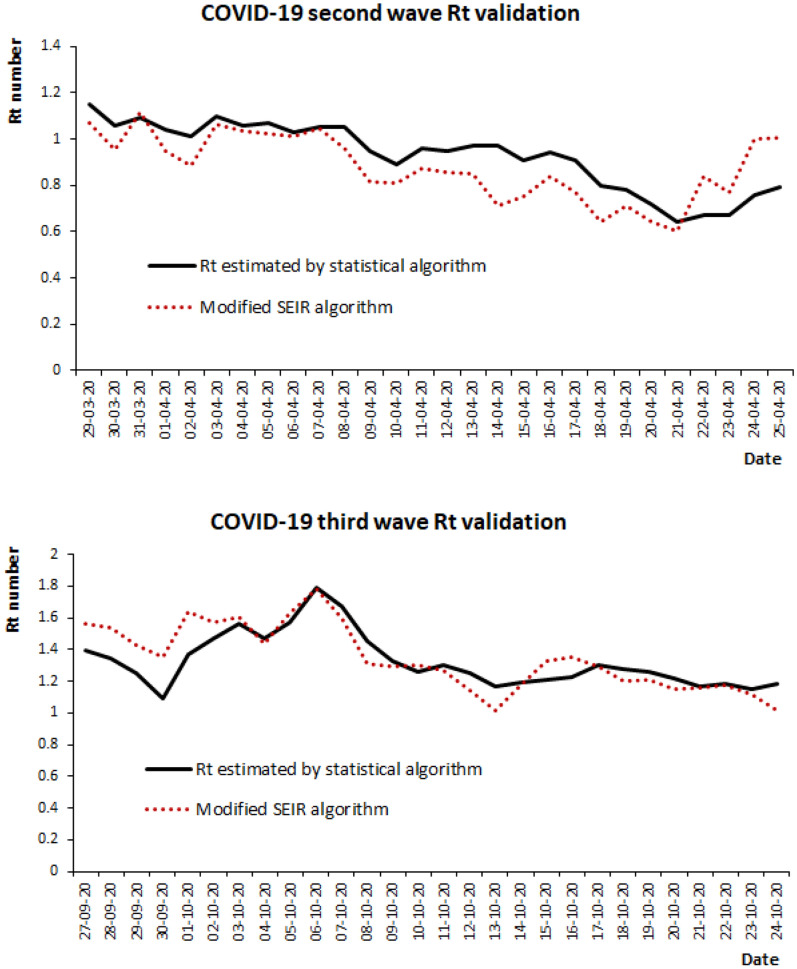
COVID-19 Second and Third wave Rt validation.

Thus, to validate accurately the Rt values generated by the algorithm, these Rt estimates were corroborated by R0 values produced by the moving window SEIR model as above. A Pearson’s correlation between the Rt values estimated by the algorithm and the moving window SEIR model was r = 0.70, p < 0.001 and r = 0.81, p < 0.001 during the validation period in the second wave (Epidemiological week from 14 to 17) and third wave (Epidemiological week 40 to 44) respectively as shown in Fig. 4.

### Effects of events on the Rt values for the second COVID-19 wave

**Figure 5:**
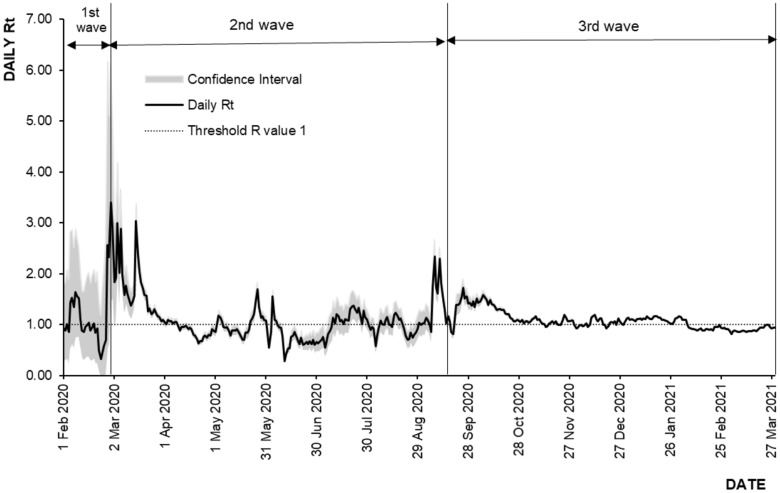
Daily time varying reproduction number, 1 February 2020–29 March 2021, Malaysia.

The daily Rt values were estimated for all three COVID-19 waves from 1 February 2020 to 29 March 2021 as shown in Table 1. There was a total of 423 values estimated for the study period as shown in Fig. 5, the highest Rt’s estimated was 3.40 (95% CI 1.47, 6.14) on 29 February 2020, and the lowest was 0.28 (95% CI 0.23, 0.33) on 11 Jun 2020.

**Table 1:** Characteristics of estimated Rt across each COVID-19 wave, Malaysia.

Variables	First wave	Second wave	Third wave*
Time period	25/1/2020 to 26/2/2020	27/2/2020 to 19/9/2020	20/9/2020 to 29/3/2021
Duration	33 days	206 days	191 days
Highest Rt value	1.64 (95% CI 0.75, 2.88)	3.40 (95% CI 1.47, 6.14)	1.72 (95% CI 1.54, 1.90)
Lowest Rt value	0.32 (95% CI 0.01, 1.19)	0.28 (95% CI 0.23, 0.33)	0.81 (95% CI 0.80, 0.82)
Range	0.32 to 1.64	0.28 to 3.40	0.81–1.72
Median Rt value	0.95	1.00	1.06
Rt trend	Reduction 2.80% per day (95% CI 2.80, 2.84)	Reduction 0.43% per day (95% CI 0.32, 0.60)	Reduction 0.24% per day (95% CI 0.24, 0.25)

*Rt estimated by excluding detention cases.

For the first wave, a total of 26 Rt values were estimated with the highest and lowest values were estimated at Rt = 1.64 (95% CI 0.75, 2.88) and Rt = 0.32 (95% CI 0.01, 1.19) on 8 February 2020 and 23 February 2020 respectively. The range was between Rt = 0.32 to 1.64 with a median Rt = 0.95. The median daily Rt reduction of 2.80% (95% CI 2.80, 2.84) was observed from 25 January 2020 to 26 February 2020 (first wave).

As for the second wave, a total of 206 Rt values were estimated with the highest and lowest values were estimated at 3.40 (95% CI 1.47, 6.14) and 0.28 (95% CI 0.23, 0.33) on 29 February 2020 and 11 June 2020 respectively. The range was between Rt = 0.28 to 3.40 with a median Rt = 1.00. The median daily Rt reduction of 0.43% (95% CI 0.32, 0.60) was observed from 27 February 2020 to 19 September 2020 (second wave).

During the second wave, there was a total of six events observed, a mass gathering in Sri Petaling (disease propagation event) and another five MCO phases (disease control events) shown in Table 2.

**Table 2:** Estimated Rt values across various events during the second wave of COVID-19.

	Sri Petaling event	MCO Phase 1	MCO Phase 2	MCO Phase 3	CMCO	RMCO
Time period	27/2/2020 to 15/3/2020	18/3/2020 to 31/3/2020	1/4/2020 to 14/4/2020	15/4/2020 to 28/4/2020	29/4/2020 to 9/6/2020	10/6/2020 to 31/08/2020
Duration	18 days	14 days	14 days	14 days	42 days	83 days
Highest Rt value	3.40 (95%CI 1.47–6.14)	1.84 (95% CI 1.70, 1.98)	1.10 (95% CI 1.04, 1.17)	0.94 (95% CI 0.88, 1.00)	1.69 (95% CI 1.56, 1.83)	1.37 (95% CI 1.03, 1.61)
Lowest Rt value	1.37 (95% CI 1.12, 1.64)	1.06 (95% CI 1.00–1.12)	0.89 (95% CI 0.84, 0.95)	0.64 (95% CI 0.58, 0.70)	0.55 (95% CI 0.49, 0.62)	0.28 (95% CI 0.23, 0.33)
Range	1.37 to 3.40	1.06 to 1.84	0.89 to 1.10	0.64 to 0.94	0.55 to 1.69	0.28 to 1.37
Median Rt value	1.95	1.25	1.02	0.78	0.94	0.93
Rt trend	Increase 2.82% per day (95% CI 2.50, 2.90)	Reduction 5.27% per day (95% CI 4.76, 5.80)	Reduction 1.00% per day (95% CI 0.95, 1.02)	Reduction 1.1% per day (95% CI 0.94, 1.29)	Increase 0.43% per day (95% CI 0.41, 0.44)	Increase 0.48% per day (95% CI 0.39, 0.58)
Reduction compared to highest Rt 3.40*	–	63.2%	70.0%	77.1%	72.5%	72.6%

*Highest estimated Rt during the second wave of COVID-19 was 3.4 due to Sri Petaling gathering.

As for the mass gathering event in Sri Petaling Gathering, a total of 18 Rt values were estimated with the highest and lowest values were estimated at Rt = 3.40 (95% CI 1.47, 6.14) and Rt = 1.37 (95% CI 1.12, 1.64) on 29 February 2020 and 12 March 2020 respectively. The range was between Rt = 1.37 to 3.40 with a median Rt = 1.95. The median daily Rt increase of 2.82% (95% CI 2.50, 2.90) was observed during this event.

During the MCO phase 1, a total of 14 Rt values were estimated with the highest and lowest values were estimated at Rt = 1.84 (95% CI 1.70, 1.98) and Rt = 1.06 (95% CI 1.00, 1.12) on 18 March 2020 and 30 March 2020 respectively. The range was between Rt = 1.06 to 1.84 with a median Rt = 1.25. The median daily Rt reduction of 5.27% (95% CI 4.76, 5.80) was observed during this event.

As for MCO phase 2, a total of 14 Rt values were estimated with the highest and lowest values were estimated at Rt = 1.10 (95% CI 1.04, 1.17) and Rt = 0.89 (95% CI 0.84, 0.95) on 3 April 2020 and 10 April 2020, respectively. The range was between Rt = 0.89 to 1.10 with a median Rt = 1.02. The median daily Rt reduction of 1.00% (95% CI 0.95, 1.02) was observed during this event.

For the MCO phase 3, a total of 14 Rt values were estimated with the highest and lowest values were estimated at Rt = 0.94 (95% CI 0.88, 1.00) and Rt = 0.64 (95% CI 0.58, 0.70) on 16 April 2020 and 21 April 2020 respectively. The range was between Rt = 0.64 to 0.94 with a median Rt = 0.78. The median daily Rt reduction of 1.10% (95% CI 0.94, 1.29) was observed during this event.

While for the CMCO phase, a total of 42 Rt values were estimated with the highest and lowest values were estimated at Rt = 1.69 (95% CI 1.56, 1.83) and Rt = 0.55 (95% CI 0.49, 0.62) on 26 May 2020 and 2 June 2020 respectively. The range was between Rt = 0.55 to 1.69 with a median Rt = 0.94. The median daily Rt increase of 0.43% (95% CI 0.41, 0.44) was observed during this event.

Finally, during the RMCO phase, a total of 83 Rt values were estimated with the highest and lowest values were estimated at Rt = 1.37 (95% CI 1.03, 1.61) and Rt = 0.28 (95% CI 0.23, 0.33) on 22 July 2020 and 11 June 2020 respectively. The range was between Rt = 0.28 to 1.37 with a median Rt = 0.93. The median daily Rt increase of 0.48% (95% CI 0.39, 0.58) was observed during this event.

**Figure 6:**
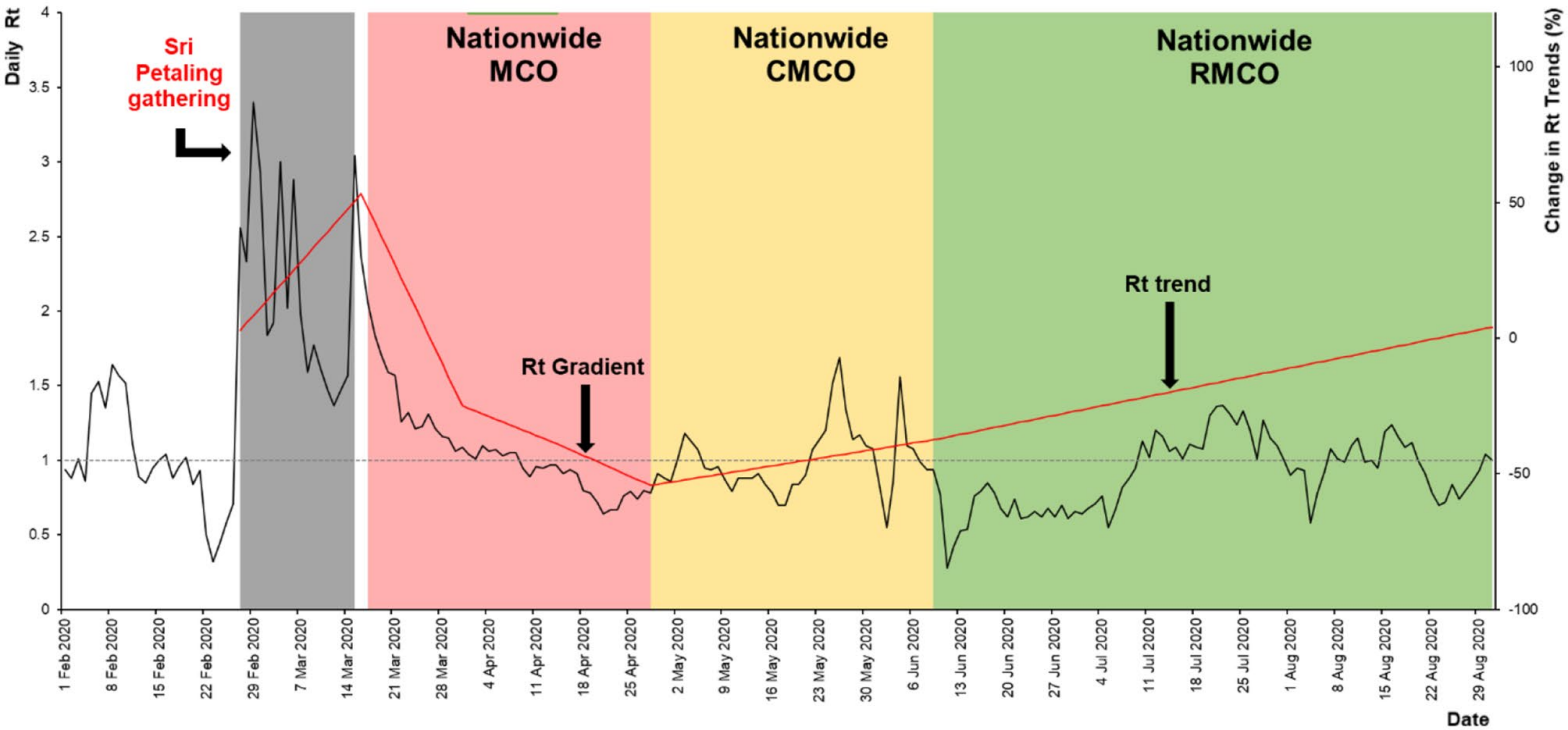
Estimated Rt during the propagation and control events, second wave of COVID-19, 1 February 2020 to 31 August 2020, Malaysia.

The highest estimated Rt during the second wave of COVID-19 was 3.40, this was for the Sri Petaling event. Following this event, the Rt started to drop for each phase of the MCO to reach a median value of Rt = 0.93 for the RMCO. The reduction from the highest estimated Rt during the Sri Petaling event (Rt = 3.40) as a result of the various phases of MCO for the second wave of COVID-19 ranged from 63.2% to 77.1%. Figure 6 shows the estimated Rt for the propagation and control events for the second wave of COVID-in Malaysia.

**Figure 7:**
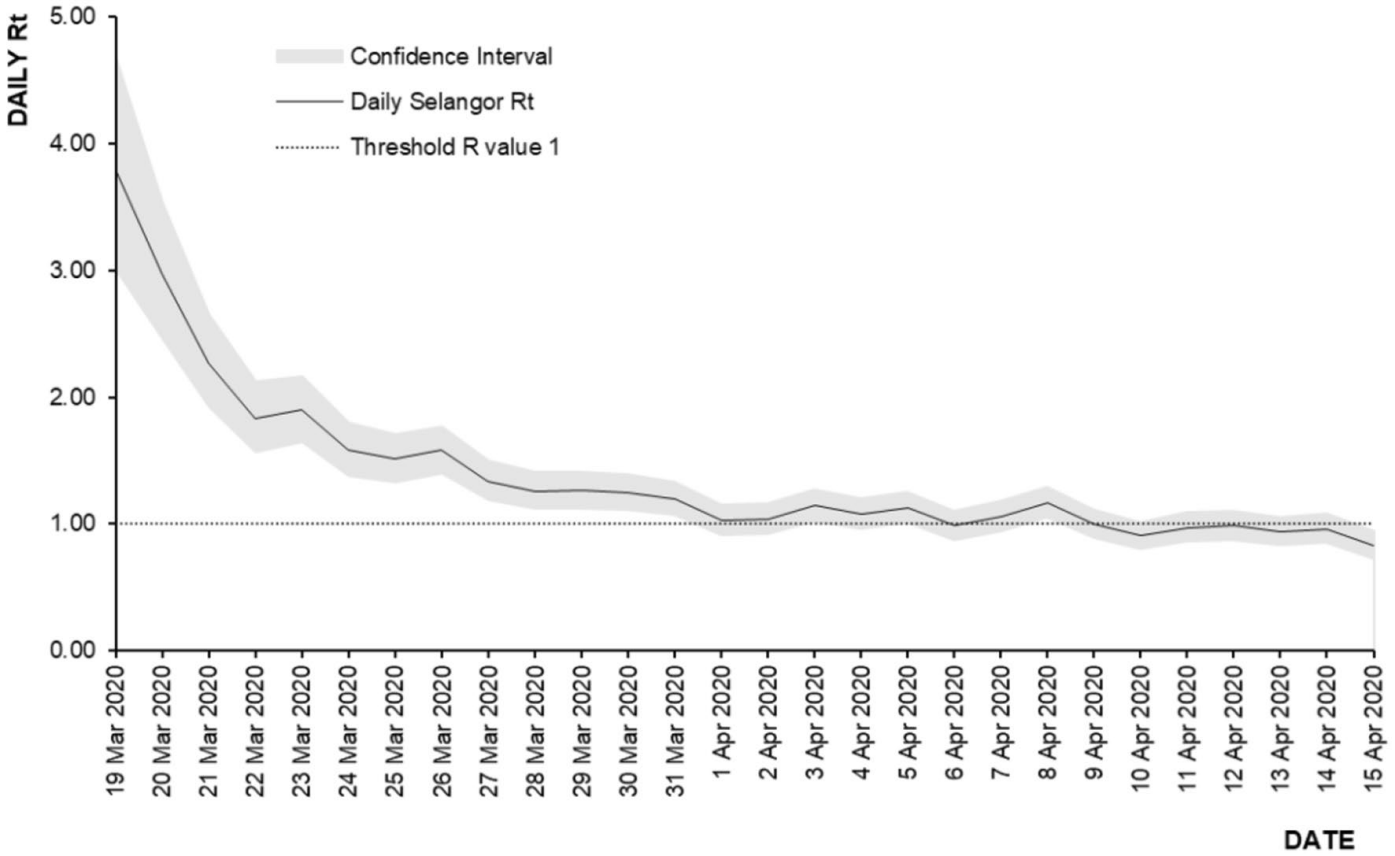
Rt trend following Sri Petaling mass gathering event, 19 March 2020 to 15 April 2020, Selangor.

The highest recorded Rt in Selangor State following the Sri Petaling Mass Gathering event was Rt = 3.80 (95% CI 3.01–4.68) on 19 March 2020 as shown in Fig. 7. Following this event, the Rt started to reduce daily by 6.30% to reach Rt = 0.83 (95% CI 0.73–0.94) by 15 April 2020 following the institution of the MCO phase 1 and 2.

### Effects of events on the Rt values for the third COVID-19 wave

During the third wave, a total of 191 Rt values were estimated with the highest and lowest values were estimated at 1.72 (95% CI 1.54, 1.90) and 0.81 (95% CI 0.80, 0.81) on 25 September 2020 and 3 March 2021 respectively. The range was between Rt = 0.81–1.72 with a median Rt = 1.06. The median daily Rt reduction of 0.24% (95% CI 0.24, 0.25) was observed from 20 September 2020 to 29 March 2021.

As for the third wave (as of 29 March 2021), there was a total of three events observed of which one was a propagation event which was a mass gathering following electoral processes in Sabah and the others were MCO and MCO with conditional MCO (CMCO) as shown in Table 3.

**Table 3:** Estimated Rt values across various events for the third wave of COVID-19.

	Sabah electoral process	MCO	MCO with CMCO
Time period	12/9/2020 to 10/10/2020	14/10/2020 to 4/2/2021	5/2/2021 to 29/03/2021
Duration	29 days	114 days	53 days
Highest Rt value	1.72 (95% CI 1.54, 1.90)	1.34 (95% CI 1.29, 1.39)	1.00 (95% CI 0.98, 1.02)
Lowest Rt value	0.96 (95% CI 0.79, 1.15)	0.92 (95% CI 0.90, 0.94)	0.81 (95% CI 0.80, 0.82)
Range	0.96 to 1.72	0.92 to 1.34	0.81 to 1.00
Median Rt value	1.42	1.09	0.91
Rt Trend	Increase 1.40% per day (95%CI 1.00,1.90)	Decrease 0.04% per day (95% CI 0.02, 0.06)	Increase 0.01% per day (95% CI 0.01, 0.03)
Reduction compared to Rt 1.72*	–	37.0%	47.0%

Highest estimated Rt for the third wave of COVID-19 was 1.72 due to mass gathering in the Sabah electoral process.

While for the mass gathering following the electoral process in Sabah, a total of 29 Rt values were estimated with the highest and lowest values were estimated at Rt = 1.72 (95% CI 1.54, 1.90) and Rt = 0.96 (95% CI 0.79, 1.15) on 25 September 2020 and 16 September 2020 respectively. The range was between Rt = 0.96 to 1.72 with a median Rt = 1.42. The median daily Rt increase of 1.40% (95% CI 1.00, 1.90) was observed in this event.

For the MCO, a total of 114 Rt values were estimated with the highest and lowest values were estimated at Rt = 1.34 (95% CI 1.29, 1.39) and Rt = 0.92 (95% CI 0.90, 1.34) on 14 October 2020 and 1 December 2020 respectively. The range was between Rt = 0.92 to 1.34 with a median Rt = 1.09. The median daily Rt reduction of 0.04% (95% CI 0.02, 0.06) was observed in this event.

While for the MCO with CMCO phase, a total of 53 Rt values were estimated with the highest and lowest values were estimated at Rt = 1.00 (95% CI 0.98, 1.02) and Rt = 0.81 (95% CI 0.80, 0.82) on 25 March 2021 and 3 March 2020 respectively. The range was between Rt = 0.81 to 1.00 with a median Rt = 0.91. The median daily Rt increase of 0.01% (95% CI 0.01, 0.03) was observed during this event.

**Figure 8:**
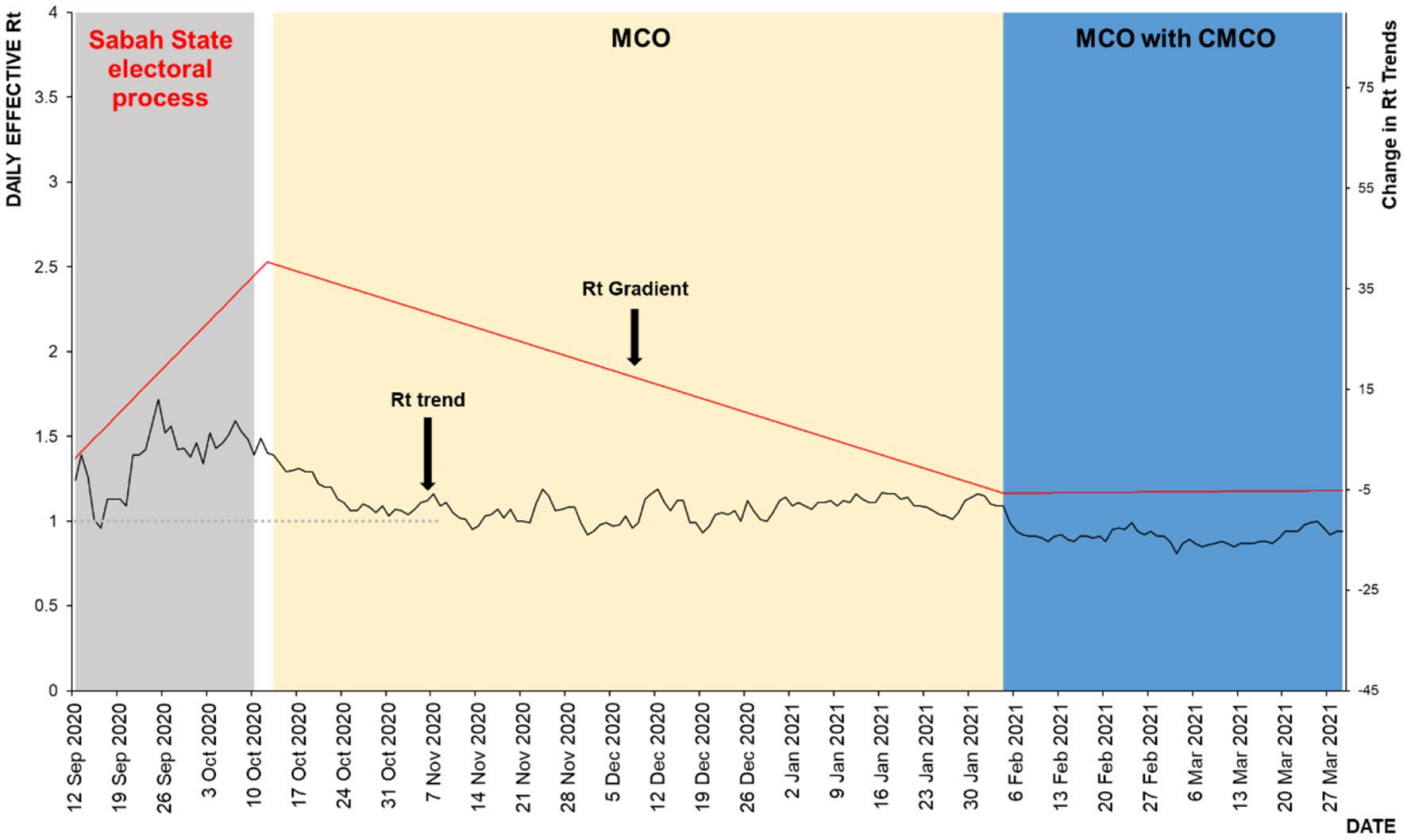
Estimated Rt for the propagation and control events, third wave of COVID-19, 12 September 2020 to 29 March 2021, Malaysia.

**Figure 9:**
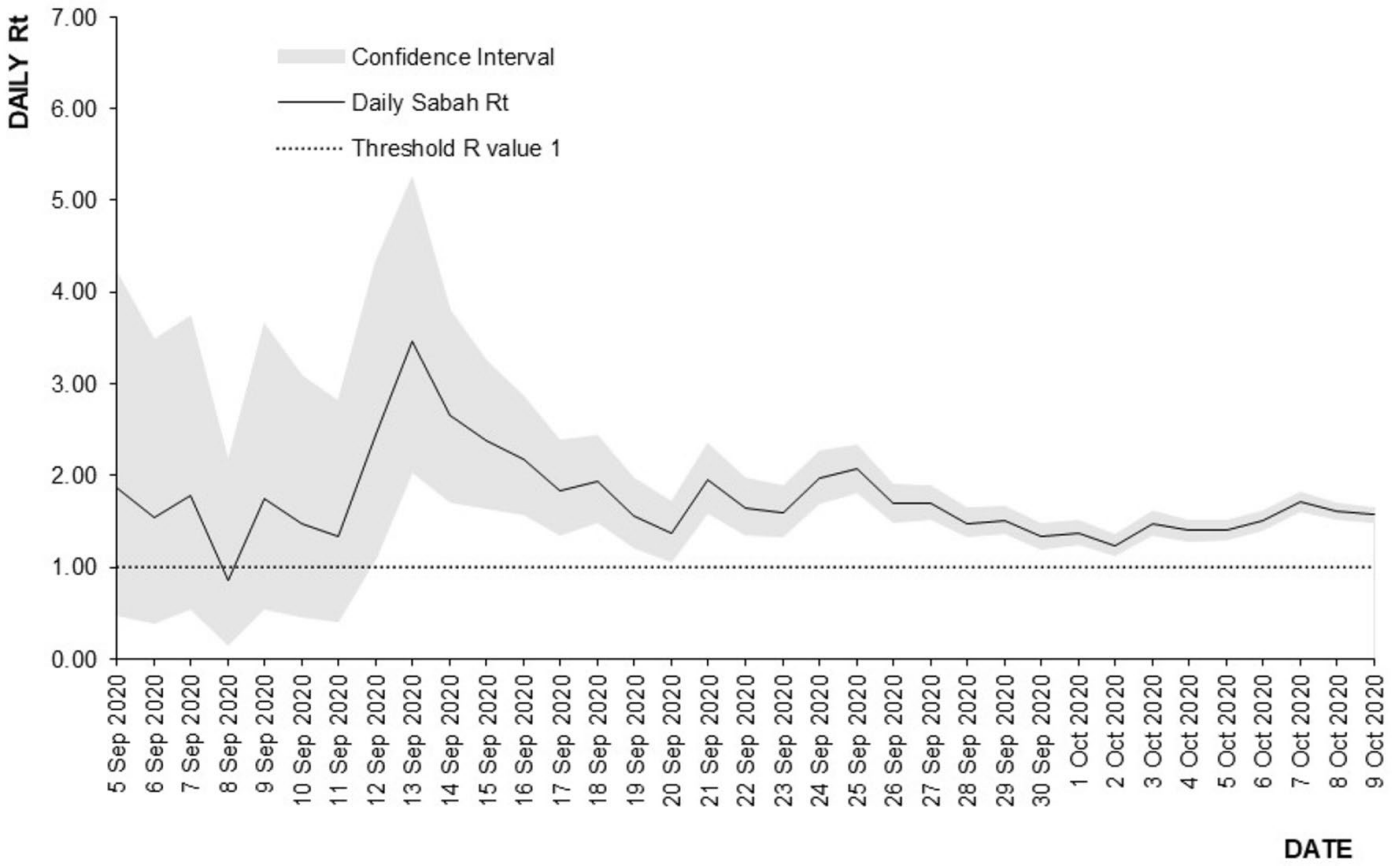
Rt trend following Sabah electoral event, 5 September 2020 to 9 October 2020, Sabah.

The highest estimated Rt for the third wave of COVID-19 was Rt = 1.72, this was due to the mass gathering following the electoral process. Following this event, the Rt started to drop to reach a median value of Rt = 0.91 for the MCO with CMCO phase. The reduction from the highest estimated Rt during the Sabah electoral process (Rt = 1.72) as a result of the various phases of MCO for the third wave of COVID-19 ranged from 37.0 to 47.0%. Figure 8 shows the estimated Rt for the propagation and control events for the third wave of COVID-in Malaysia. The highest recorded Rt in Sabah state following the Sabah electoral process was Rt = 3.46 (95% CI 2.04–5.25) on 13 September 2020 as shown in Fig. 9. Following this event, the Rt started to reduce daily by 4.1% to reach Rt = 1.57 (95% CI 1.49–1.65) by 9 October 2020 following the institution of the targeted CMCO.

## Discussion

Challenges faced by health systems in monitoring COVID-19 have resulted in failures to contain the spread of the pandemic^30^. This is because of the novel nature of this disease, the absence of baseline data as a reference and the explosive nature of this pandemic. Besides this, the indicators used to monitor the progression of the COVID-19 pandemic such as case incidence, doubling time and growth rate are unable to provide accurate and sensitive monitoring of the pandemic as these indicators were unable to determine the disease transmissibility^16,17^. However, literature suggests that the most effective way to measure disease transmissibility is by determining its R value. This is because R values are able to provide sensitive indicators that reflects the disease transmissibility and provide time-dependent variations in the transmission potential and progression of the pandemic^16,17^.

There are several methods to estimate the Rt values that are based on fitting mechanistic or deterministic transmission models to epidemiological data^18,31^. More specifically these methods are Exponential growth rate, Maximum Likelihood, Markov Chain Monte Carlo, Next-Generation Matrix Approach, Epidemic curve model, and compartmental model methods^32^. However, estimating the Rt using these methods are complex, time consuming, and may cause challenges to provide real time daily Rt estimates for multiple geographical areas. In addition, developing these model requires advance statistical capabilities, and furthermore, it may not be possible to be carried out at the ground level where the outbreak is occurring as it requires complex computations for each Rt value generated^31^.

In this paper we are able to generate real time daily COVID-19 Rt values for Malaysia at national and state levels by adopting and modifying the Rt estimation algorithm developed by Anne Cori et al.^18^. The Rt algorithm used in this paper was simple to implement and is more flexible than using the above mention methods or fitting mechanistic or deterministic transmission model methods. In addition, this algorithm is able to produce similarly accurate Rt values quickly and efficiently, compared to those complex methods as above. Furthermore, we validated this algorithm for its accuracy, as to date there has not been any Rt values generated for the COVID-19 pandemic in Malaysia that has been statistically validated using Bayesian inferential methods. In this study the Rt values generated was validated using Bayesian methods as it was the most appropriate validation approach, allowing for model validation under conditions of parameter uncertainty and with limited testing data^33^. The results of the validation reported highly correlated values that suggest that the Rt values generated by the algorithm were accurate. Validation using the above method ensures that the Rt values generated using the algorithm in this study are valid, reliable, context-specific and represents the COVID-19 disease trends in Malaysia. With this validated algorithm, it can be concluded that the generated Rt values are sensitive indicators that are essential for monitoring and controlling the COVID-19 pandemic in Malaysia.

Since the Rt estimation algorithm in this study generated valid daily Rt values that are essential in monitoring and assisting in the control of the COVID-19 pandemic in Malaysia, we developed an automated web application that uses the validated algorithm to estimate daily real-time Rt values for Malaysia. In the absence of a real-time COVID-19 transmissibility monitoring system in Malaysia, this automated application assists the surveillance system by providing daily Rt values in real time to the health authorities and the public. Providing regular COVID-19 Rt updates allows for evidence-based adjustment of control measures, enables the assessments of the effectiveness of control measures and assists in the accurate forecasting of COVID-19 case trends in Malaysia. In addition to this, continuous access of Rt values to the public via the *MySejahtera* mobile application and the MOH COVID-19 website would increase public awareness and compliance to the COVID-19 Standard Operating Procedures (SOPs). The Rt estimation algorithm is an important intervention developed in this study which assists in the control of the COVID-19 pandemic in Malaysia.

Being able to generate daily validated COVID-19 Rt values, we examined the outbreak propagation and control events in Malaysia by measuring the changes and progression of the daily estimated Rt values as a result of these events. We found that in Malaysia, mass gathering events were one of the main drivers for the COVID-19 pandemic. For example, the Sri Petaling mass gathering in February 2020 and the electoral process in Sabah in September 2020 were catalysts for the second and third COVID-19 waves in Malaysia respectively. Similar findings of mass gatherings events resulting in increased transmissibility of COVID-19 have been reported in India, United States, Japan^7,34,35^. In India the religious mass gatherings had resulted in a large increase in COVID-19 cases observed in March 2020^7^. While in the United States, electoral process had led to a 45% rise in daily cases in November 2020^34^. In addition, mass confinement of individual as observed in Diamond Princess cruise ship in Japan resulted in a high R value of 11 which further suggests that mass gatherings especially in confined environments are key drivers for the explosive transmission of COVID-19^35^. This can be explained by the increased risk of disease transmissions due to the large aggregation of individuals in a particular area in a given time, which allows for propagation of droplets and airborne transmission as observed during electoral process seen in Sabah^5^. In addition, this method of transmission increases especially in confined spaces over prolonged periods as observed during the Sri Petaling mass gathering. Furthermore, a lack of enforcement and compliance to Standard Operating Procedures (SOP) such as the use of face masks, hand/cough hygiene, physical distancing during these events and environmental factors such as temperature, relative humidity, pollution, solar radiation may further increase the explosive nature of the outbreak^5,36^.

The above suggestions can be explained by the findings in this study where we found that the National daily estimated Rt increased to 1.72 during the Sabah electoral process which was a mass gathering event. However, the Rt during the Sri Petaling event was much higher at 3.40 as it was a mass gathering event conducted in a confined space. In addition, due to this, the daily case numbers started to increase 1 to 4 weeks following the onset of these events.

In addition, the high Rt observed during the Sri Petaling mass gathering could also be attributed due to the event occur early on in the pandemic, whereby there was a lack of awareness among the public regarding COVID-19 resulting in poor compliance to SOPs (i.e. use of face mask, regular hand hygiene and physical distancing)^37^. Compared to the Sri Petaling event, the Rt during the Sabah electoral process was lower, as this mass gathering event did not occur in a confined area and consisted of multiple propagated small events occurring in open areas out over a longer period. Furthermore, there were already established ongoing COVID-19 control measures and SOP compliance compared to the Sri Petaling event which resulted in this difference.

To further confirm the effects of these events on the transmissibility of the COVID-19 pandemic in Malaysia, we found that the rise in Rt values were higher in the states where the events occurred as compared to the National Rt during the similar period. Where in the Rt for Selangor in the second wave and Sabah in the third wave was 3.80 and 3.46 respectively which was significantly higher than the National Rt of 3.40 and 1.72 during the second and thirds waves respectively. This finding shows that the increase in Rt was attributed to the mass gathering events as evident by the increase of Rt at the states where these events occurred. This supports our suggestion that these mass gathering events were among the factors responsible for the second and third COVID-19 waves in Malaysia.

In response to the surge of cases as a result of these mass gathering events, the Government of Malaysia instituted Non Pharmaceutical Interventions such as the MCO^14^. Instituting the MCO has been shown to be an effective measure to control the transmission of COVID-19 as these restrictions limit physical contact by avoiding unnecessary movements of population^15^. In this study we found that during the second wave of COVID-19 pandemic in Malaysia the MCO phase was able to reduce the Rt value by 63.2% to 77.1%. Similarly, a reduction of 37.0% to 47.0% was observed during the MCO and CMCO phase in the third wave. Similar findings have been observed in Canada, China, Mexico and Niger whereby during the lockdown periods, the R values were reduced by 73% to 82% compared to pre-lockdown levels^38^.

While movement restrictions are effective mechanism to control the COVID-19 pandemic, however its implementation has an effect on social and economic situation of the nation^39^. In order to strike a balance between life and livelihood, the MCO allows for adjustments to its intensity by a step up and stepping down approach for example the MCO, CMCO and RMCO^40^. In this paper, we found that higher MCO stringency levels resulted in a larger reduction in daily Rt values, wherein we found the MCO phases resulted in a daily Rt reduction ranging from 1.10 to 5.27%. Compared to MCO, the CMCO and RMCO resulted in an increase in the daily Rt at 0.43% and 0.48% respectively. While the there was an increase in daily Rt during the CMCO and RMCO phases with median Rt of 0.94 and 0.93 respectively compared to the Rt of 3.4 during the Sri Petaling event, our results suggest that by adjusting MCO stringency level it is possible to balance the need to control the pandemic while ensuring the continuation of social and economic activities. Similarly a study in China found an inverse relation between the severity of movement restriction and Rt values, wherein a 20 to 60% mobility restriction resulted in Rt reduction of 25 to 50% respectively^41^.

This study has several strengths, first, we developed a web-based automated system that operates on Rt algorithms that was validated using computationally advantageous approaches (i.e. SEIR models and the existing Bayesian framework) to produce continuous, real-time Rt values which are context-specific for Malaysia at a national and state level. To date, the validation approach used has not been done in Malaysia as of this paper. Second, the Rt algorithm used in this study is an easier to implement, more flexible method that accounts for the uncertainty of the serial interval distribution which is sensitive to the size of sliding windows. Third, to ensure the Rt values generated truly reflect the community transmissibility of COVID-19 in Malaysia, we excluded detention cases from the daily case incidence during the third wave, as during this period there were clusters involving detention centres reported daily.

The limitations of this paper include firstly, data limitations due to surveillance effects such under reporting and late reporting due to delay in testing. In addition, inaccuracies of testing protocol resulting in false positive or negative results, however this is minimal as confirmation of cases was test using PCR test. Second, limitation of methodology of estimating Rt, where in the Rt algorithm in this study does not include all the factors (i.e. compliance to SOP, ventilation and environmental factors) potentially driving the spread of COVID-19’. Future research should aim to use the current models with appropriate modification/adjustment, to account for the effects of vaccinations (i.e. variation in vaccine efficacy, vaccine rollout rate) and introduction of other variants.

## Conclusion

The findings of our study confirmed that the Rt estimation algorithm was able to generate validated daily Rt values at the National and State level for Malaysia. These values were able to provide accurate and sensitive information on the disease transmissibility, transmission potential, disease progression and forecast in a timely fashion for the control of the COVID-19 pandemic in Malaysia. This paper has shown the effects of mass gathering and movement control measures on the transmissibility of the COVID-19 outbreak in Malaysia. In addition, we provide strong evidence that mass gathering events were one of the important drivers and contributed to towards the spread of COVID-19 in Malaysia, especially mass gathering in confined areas which increases the disease transmission significantly compared to open areas. However, the spread of disease can also be fueled by noncompliance to SOPs (i.e. improper use of face mask, poor practice of physical distancing, poor hand hygiene and cough ethics), poor ventilation and environmental factors (i.e. temperature, relative humidity, pollution, solar radiation)^42^. Furthermore, we provided evidence that the MCOs were able to reduce the daily Rt values, especially MCOs with higher stringency levels would correspondingly result in decreasing Rt values. Finally, we demonstrated the applicability and utilization of the Rt estimation algorithm in this paper in assisting both the health authorities and the public to monitor and control the COVID-19 pandemic in Malaysia.

## Supplementary Information

Supplementary Information.

## Data Availability

All data used in this study were open source data available at MOH official website http://covid-19.moh.gov.my/. The generated Rt values are attached as separate file.
