# Supplementary material for: The effects of super spreading events and movement control measures on the COVID-19 pandemic in Malaysia
Source: Sci Rep. 2022 Feb 9;12:2197. doi: 10.1038/s41598-022-06341-1 (PMC8828893; doi:10.1038/s41598-022-06341-1)
Supplement: Supplementary file 1 — Supplementary Information. [file 41598_2022_6341_MOESM1_ESM.docx]

# **Appendix 1**

**Table 1. Test of Normality**

| **Tests of Normality** | | | | | | |
| --- | --- | --- | --- | --- | --- | --- |
|  | Kolmogorov-Smirnov^a^ | | | Shapiro-Wilk | | |
|  | Statistic | df | Sig. | Statistic | df | Sig. |
| **Second wave**  (Epidemiological week from 14 to 17) |  |  |  |  |  |  |
| Rt_algorithm | .139 | 28 | **.174** | .926 | 28 | **.050** |
| Rt_SEIR | .122 | 28 | **.200^*^** | .958 | 28 | **.311** |
| **Third wave**  (Epidemiological week 40 to 43) |  |  |  |  |  |  |
| Rt_algorithm | .155 | 28 | **.084** | .923 | 28 | **.052** |
| Rt_SEIR | .132 | 28 | **.200^*^** | .951 | 28 | **.215** |
| *. This is a lower bound of the true significance. | | | | | | |
| a. Lilliefors Significance Correction | | | | | | |


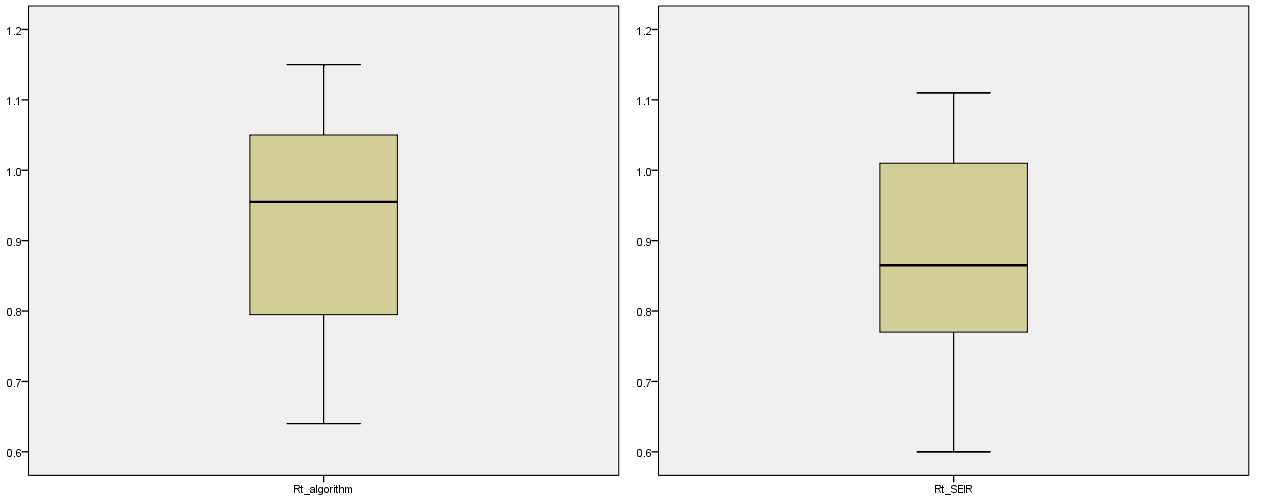


Figure 1. Test for outliers, box plot for Rt estimates from algorithm and SEIR model, second wave (Epidemiological week from 14 to 17)


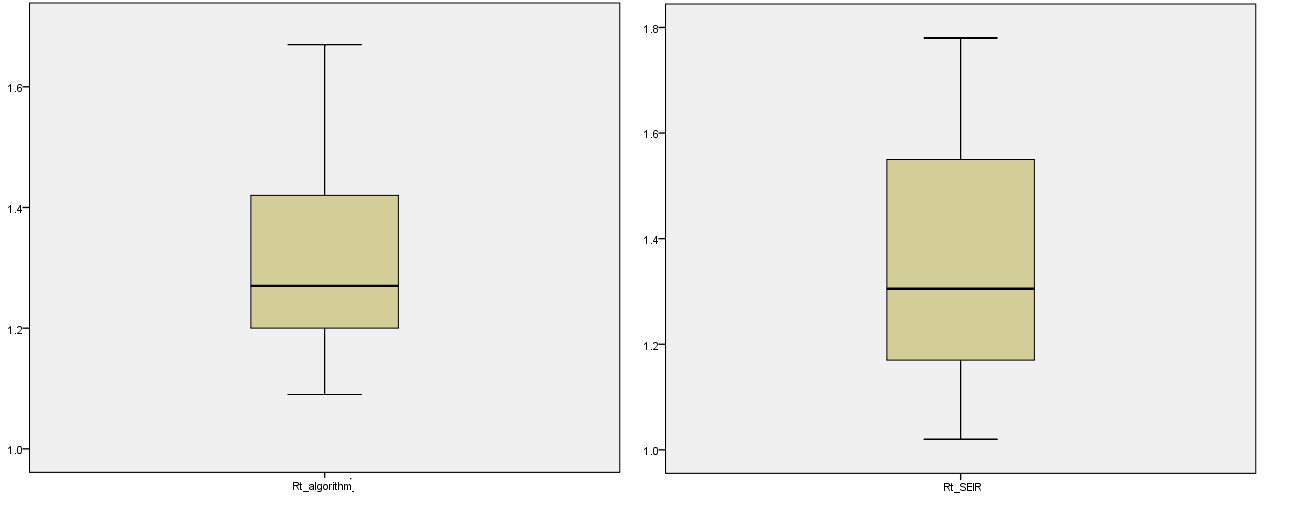


Figure 2. Test for outliers, box plot for Rt estimates from algorithm and SEIR model, third wave (Epidemiological week 40 to 43)

**Table 2. Test for Homoscedasticity**

| **Test for Homoscedasticity** | | | | |
| --- | --- | --- | --- | --- |
|  | | | Standardized Predicted Value | Absolute Residual Value |
| **Second wave (Epidemiological week from 14 to 17)** | | |  |  |
| Spearman's rho | Standardized Predicted Value | Correlation Coefficient | 1.000 | -.196 |
|  |  | Sig. (2-tailed) | . | **.316** |
|  |  | N | 28 | 28 |
|  | Absolute Residual Value | Correlation Coefficient | -.196 | 1.000 |
|  |  | Sig. (2-tailed) | .316 | . |
|  |  | N | 28 | 28 |
| **Third wave (Epidemiological week 40 to 43)** | | |  |  |
| Spearman's rho | Standardized Predicted Value | Correlation Coefficient | 1.000 | .390 |
|  |  | Sig. (2-tailed) | . | **.050** |
|  |  | N | 28 | 28 |
|  | Absolute Residual Value | Correlation Coefficient | .390^*^ | 1.000 |
|  |  | Sig. (2-tailed) | .040 | . |
|  |  | N | 28 | 28 |

*. Correlation is significant at the 0.05 level (2-tailed)

**
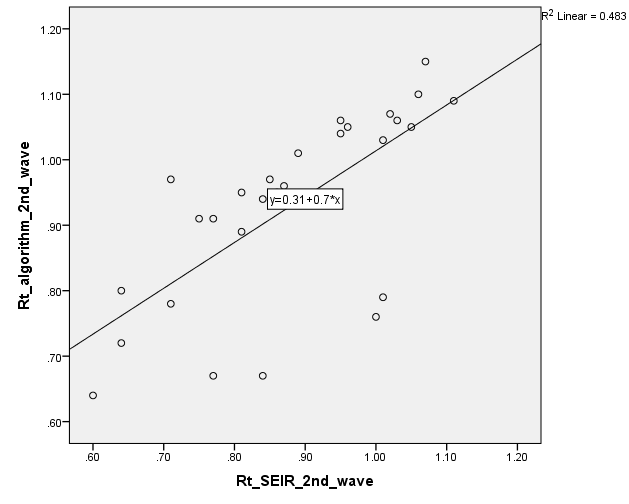
**

**
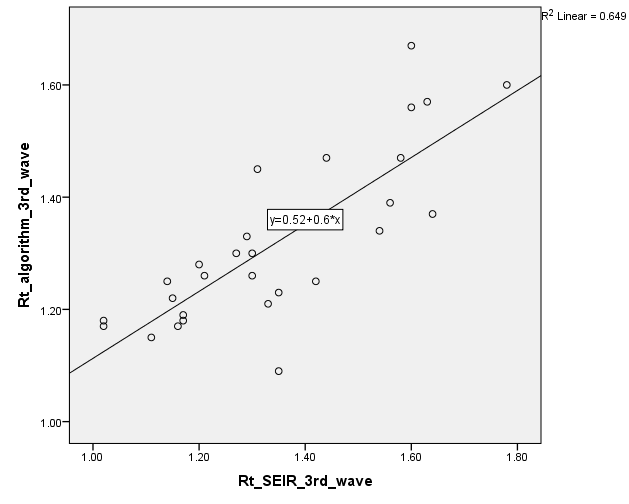
**

Figure 3. Test for linearity, Scatter for Rt estimates from algorithm and SEIR model, second wave (Epidemiological week from 14 to 17) and third wave (Epidemiological week 40 to 43)
